# Elevated serum SDMA and ADMA at hospital admission predict in-hospital mortality of COVID-19 patients

**DOI:** 10.1038/s41598-021-89180-w

**Published:** 2021-05-10

**Authors:** Juliane Hannemann, Paul Balfanz, Edzard Schwedhelm, Bojan Hartmann, Johanna Ule, Dirk Müller-Wieland, Edgar Dahl, Michael Dreher, Nikolaus Marx, Rainer Böger

**Affiliations:** 1 Institute of Clinical Pharmacology and Toxicology, University Medical Center Hamburg-Eppendorf, Martinistr. 52, 20246 Hamburg, Germany; 2 Institute DECIPHER, German-Chilean Institute for Research on Pulmonary Hypoxia and Its Health Sequelae, Hamburg, Germany; 3 Department of Cardiology, Angiology and Intensive Care Medicine, Medical Clinic I, University Hospital Aachen, Aachen, Germany; 4 German Centre for Cardiovascular Research (DZHK), partner site Hamburg/Kiel/Lübeck, Hamburg, Germany; 5 Institute of Pathology, University Hospital Aachen, Aachen, Germany; 6 RWTH centralized Biomaterial Bank (RWTH cBMB), Medical Faculty, RWTH Aachen University, Aachen, Germany; 7 Department of Pneumology and Intensive Care Medicine, Medical Clinic V, University Hospital Aachen, Aachen, Germany

**Keywords:** Biomarkers, Viral infection, Risk factors

## Abstract

COVID-19 is a disease with a variable clinical course ranging from mild symptoms to critical illness, organ failure, and death. Prospective biomarkers may help to predict the severity of an individual’s clinical course and mortality risk. We analyzed asymmetric (ADMA) and symmetric dimethylarginine (SDMA) in blood samples from 31 patients hospitalized for COVID-19. We calculated associations of ADMA and SDMA with mortality and organ failure, and we developed a predictive algorithm based upon these biomarkers to predict mortality risk. Nine patients (29%) experienced in-hospital death. SDMA and ADMA serum concentrations were significantly higher at admission in COVID-19 patients who died than in survivors. Cut-offs of 0.90 µmol/L for SDMA (AUC, 0.904, *p* = 0.0005) and 0.66 µmol/L for ADMA (AUC, 0.874, *p* = 0.0013) were found in ROC analyses to best discriminate both subgroups of patients. Hazard ratio for in-hospital mortality was 12.2 (95% CI: 2.2–31.2) for SDMA and 6.3 (1.1–14.7) for ADMA above cut-off. Sequential analysis of both biomarkers allowed discriminating a high-risk group (87.5% mortality) from an intermediate-risk group (25% mortality) and a low-risk group (0% mortality). Elevated circulating concentrations of SDMA and ADMA may help to better identify COVID-19 patients with a high risk of in-hospital mortality.

## Introduction

Infection with severe acute respiratory syndrome coronavirus 2 (SARS-CoV-2) causes COVID-19 that has evolved into a pandemic since first observed in China in late 2019. Most SARS-CoV-2-infected patients remain asymptomatic or develop mild symptoms of respiratory infection and fever. However, about 19% of infected individuals develop a severe course of the disease requiring hospitalization^1^. The disease may aggravate in a few patients into life-threatening acute respiratory failure, requiring intensive care treatment and respiratory support including extracorporeal membrane oxygenation (ECMO). Ultimately, mortality is associated with multiple factors including comorbidities like diabetes or cardiovascular diseases^2^.

During the first surge of SARS-CoV-2 infection, the individual outcome of patients has remained rather unpredictable, and multiple efforts have been undertaken to identify co-morbidities, biomarkers, and clinical or imaging scores to better predict outcome of hospitalized COVID-19 patients^3,4^. A severity score consisting of age, oxygen saturation, mean arterial pressure, blood urea nitrogen, C-reactive protein, and the international normalized ratio was shown to improve prediction of COVID-19-related in-hospital mortality^5^. Other groups proposed redefining established risk factors of cardiac disease^6^ or organ damage markers like aspartate aminotransferase and alanine aminotransferase^7^ to better predict worse outcomes in COVID-19 patients. Another risk score for prediction of mortality of hospitalized COVID-19 patients comprised age, heart rate, oxygen saturation, lactate dehydrogenase activity, pro-calcitonin (PCT) and the presence or absence of chronic obstructive pulmonary disease or congestive heart failure^8^. Conditions that affect the course of the disease and outcome of COVID-19 patients include pre-existing morbidities like obesity or type 2 diabetes^9^, evolvement of thromboembolic vascular complications^10^, acute kidney failure^11,12^, and neurovascular damage^4^. The broad spectrum of individual disease courses and outcomes calls for continued efforts to identify biomarkers for risk prediction, as the second and, meanwhile, third waves of the pandemic are ongoing and numbers of infected individuals are rapidly rising. The identification of novel risk factors or biomarkers leading to early identification of patients at high risk of dying of the SARS-CoV-2 infection might help to better target these patients to early monitoring and intervention^13^.

Dysfunctional production of nitric oxide (NO) by endothelial cells may contribute to COVID-19-associated morbidities^9–11^. Endothelial cell infection by SARS-CoV-2 may lead to endothelial inflammation and dysfunction of its paracrine functions^14^. Angiotensin-converting enzyme 2 (ACE2), an important regulator of NO release^15^, is also the host cell receptor for SARS-CoV-2^16^, thus linking SARS-CoV-2 infection and endothelial NO signaling. NO synthesis is tightly regulated^17^. Asymmetric (ADMA) and symmetric dimethylarginine (SDMA) are endogenous modulators of NO synthesis and intracellular L-arginine availability in the endothelium^18^; their circulating concentrations being dysregulated in hypoxia^19^. In addition, inhibition of NO synthesis by ADMA and SDMA may affect immune response and inflammatory reaction, as they also interfere with inducible NO synthase, an enzyme that is upregulated by inflammatory cytokines^20^.

Both metabolites have been shown to predict morbidity and mortality risk in populations with a wide range of risk: ADMA is associated with all-cause mortality in the general population^21,22^, in patients with end-stage renal disease^23^, and in patients with pre-existing cardiovascular disease^24^. SDMA is a predictor of mortality after acute ischemic stroke^25^ and in the general population. Both also help to identify high-risk patients within critically ill populations: ADMA and SDMA have been shown by our group to be predictors of mortality in patients with severe sepsis^26^. Nijveldt and co-workers proposed high circulating ADMA concentrations as risk factors of ICU mortality in critically ill patients^27^; moreover, ADMA is a marker of perioperative complications in patients undergoing major abdominal surgery^28^. These results were confirmed in a longitudinal study of ADMA metabolism in critically ill patients^29^.

We therefore retrospectively studied a cohort of consecutive COVID-19 patients hospitalized in a tertiary care medical center, of whom blood samples were stored under standardized conditions. We assessed whether ADMA and SDMA serum concentrations may help to better identify those at high risk of COVID-19-associated organ dysfunction and death.

## Results

### Baseline characteristics and clinical course of the patients

We included 31 patients (11 women, 20 men) with a mean age of 63.3 ± 17.8 years. 15 patients were primary admissions; 16 patients were referred from other hospitals (amongst them 8 with ongoing mechanical ventilation). With the exception of one, all patients had pre-existing co-morbidities. The mean duration from first symptoms to hospital admission was 6.8 ± 6.3 days. The mean duration of treatment in our medical center was 30.6 ± 27.0 days; need for oxygen insufflation varied from 0 to 97 days with a mean of 24.8 ± 29.1 days. 19 patients were treated on ICU for a mean of 34.7 ± 31.5 days. Detailed patient characteristics of this cohort are given in Table 1.

**Table 1:** Baseline characteristics of the patients.

Parameter	Total cohort	Deceased	Survived	P*
No of patients	31	9	22	n/a
Age (years)	63.3 ± 17.8	65.6 ± 9.2	62.4 ± 20.5	0.756
Sex (m/f)	20/11	5/4	15/7	1.000
BMI (kg/m^2^)	28.5 ± 5.4	26.9 ± 6.3	29.1 ± 5.0	0.322
**Pre-existing conditions, N (%)**				
Any	30 (96.8)	9 (100)	21 (95.5)	1.000
Obesity/overweight	10 (32.3)/11 (35.5)	2 (22.2)/3 (33.3)	8 (36.4)/8 (36.4)	0.417
Lung disease	12 (38.7)	6 (66.7)	6 (27.3)	0.068
Coronary artery disease	7 (22.6)	2 (22.2)	5 (22.7)	1.000
Cerebrovascular disease	5 (16..1)	2 (22.2)	3 (13.6)	0.240
Peripheral arterial disease	2 (6.5)	1 (11.1)	1 (4.5)	0.350
Congestive heart failure	4 (12.6)	1 (11.1)	3 (13.6)	1.000
Cancer	7 (19.4)	2 (22.2)	5 (22.7)	1.000
Chronic kidney disease	5 (16.1)	2 (22.2)	3 (13.6)	0.639
Diabetes mellitus	4 (12.9)	1 (11.1)	3 (13.6)	1.000
Hypertension	19 (61.3)	7 (77.8)	12 (54.5)	0.160
Current smoking	4 (12.9)	1 (11.1)	3 (13.6)	1.000
**Clinical findings at admission, N (%)**				
SOFA score	4.2 ± 4.0	7.0 ± 3.8	3.0 ± 3.5	0.004
SaO_2_ (%)	91.6 ± 4.6	89.5 ± 4.2	92.63 ± 4.5	0.090
Dyspnoea	16 (51.6)	6 (66.7)	10 (45.5)	0.270
Fever	19 (61.3)	5 (55.6)	14 (63.6)	0.466
Cough	18 (58.1)	6 (66.7)	12 (54.5)	1.000
Taste or smell dysfunction	5 (16.1)	1 (11.1)	4 (18.2)	1.000
Fatigue	11 (35.5)	2 (22.2)	9 (40.9)	0.278
**Laboratory parameters at admission**				
Leukocyte count (1/nL)	11.4 ± 8.1	18.7 ± 11.0	8.4 ± 4.0	0.003
C-reactive protein (mg/L)	104.6 ± 85.0	182.2 ± 93.1	75.1 ± 61.4	0.003
Pro-calcitonin (ng/mL)	1.81 ± 6.33	4.89 ± 10.86	0.35 ± 0.82	< 0.001
eGFR (mL/min)	80.5 ± 28.7	68.9 ± 31.6	85.3 ± 26.8	0.153
D-dimers (ng/mL)	19,711 ± 27,243	20,393 ± 21,232	18,891 ± 35,922	0.242

BMI, body mass index; eGFR, estimated glomerular filtration rate; SaO_2_, arterial oxygen saturation; SOFA, Sequential organ failure assessment score; n/a, not applicable; n.s., not significant. Data are mean ± standard deviation or N (%) if marked. *P values were calculated using Mann–Whitney U-test for continuous variables and Chi^2^ test for categorical variables.

Nine patients (29%) died in-hospital. Causes of death were multi-organ failure in five patients (16%), respiratory failure in two (6.5%), and hemorrhagic complications in two patients with respiratory failure (6.5%). Patients who survived had a mean SOFA score at admission of 3.0 ± 3.5, whereas patients who died during in-patient treatment had a mean initial SOFA score of 7.0 ± 3.8 (p < 0.001). 14 patients had ARDS (seven of them died), five patients with ARDS were treated by ECMO (all of them died), 10 patients developed a high thromboembolic burden (six of them died), 14 patients had cardiac injury (nine of them died), 11 patients had liver injury (five of them died), eight patients developed acute kidney injury (three of them died), and ten patients developed circulatory insufficiency (three of them died). Patients who died in-hospital had significantly higher leukocyte count, higher CRP, and higher PCT concentrations at admission (Table 1). D-dimer concentrations and eGFR were not significantly different between survivors and non-survivors.

### Serum concentrations of ADMA and SDMA

Mean ADMA serum concentration was significantly higher in patients referred to our hospital either with ongoing mechanical ventilation or without (0.84 ± 0.15 µmol/L and 0.66 ± 0.29 µmol/L, respectively; *p* = n.s.); it was lowest in primary admissions (0.58 ± 0.13 µmol/L; *p* = 0.010 for difference between groups in ANOVA). By contrast, there was no significant difference in mean SDMA serum concentration between patients referred to our ICU with ongoing mechanical ventilation (0.99 ± 0.40 µmol/L), patients referred without mechanical ventilation (0.79 ± 0.24 µmol/L; *p* = n.s. vs. patients with mechanical ventilation), and primary admissions (0.85 ± 0.46 µmol/L; *p* = 0.598 for difference between groups in ANOVA). ADMA concentration correlated significantly with CRP and leukocyte count, but not with PCT and eGFR (supplementary Fig. S1). SDMA concentration correlated inversely with eGFR, positively with CRP, but not with PCT nor with leukocyte count (supplementary Fig. S2).

**Figure 1:**
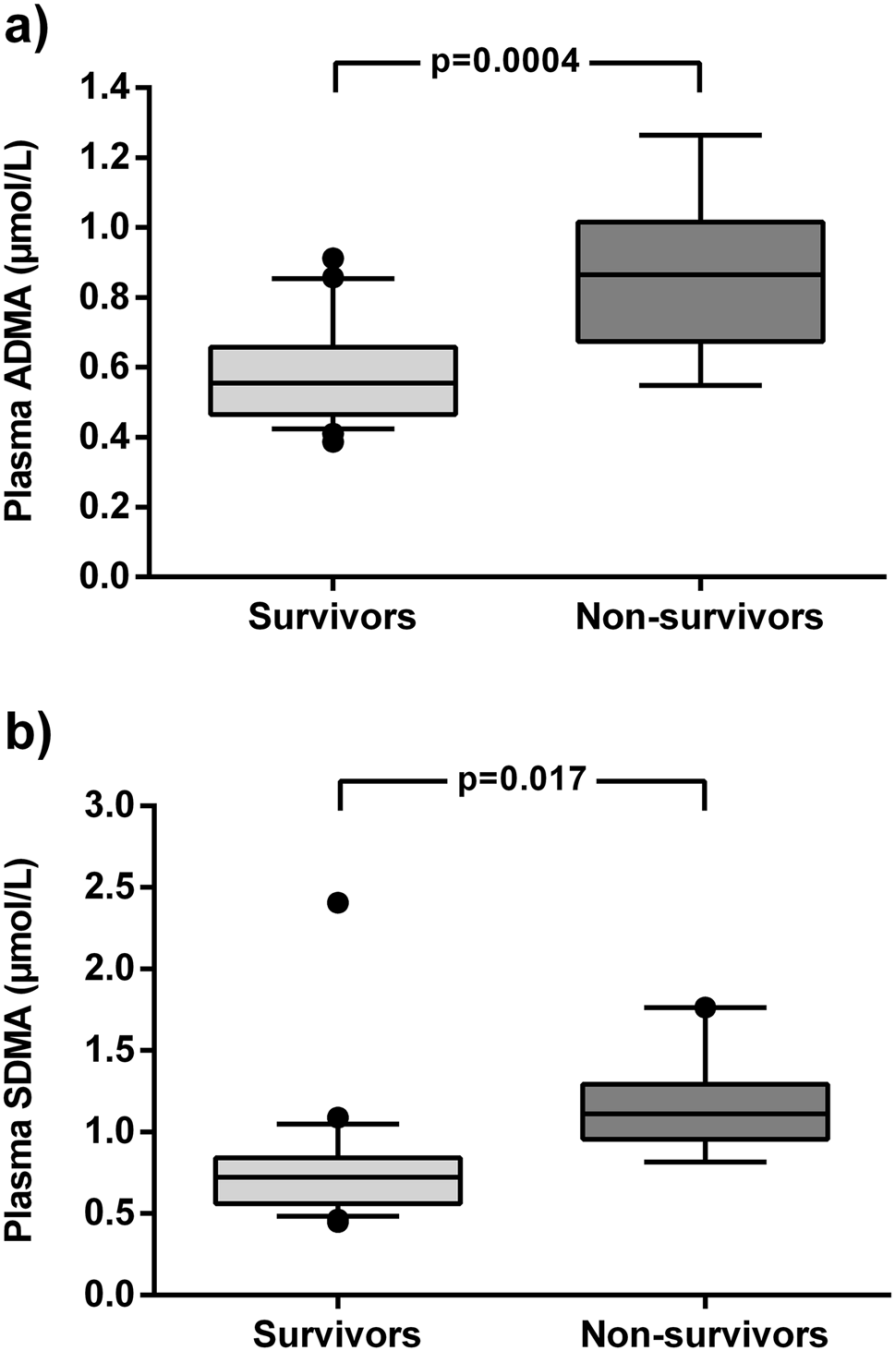
Box plots of the serum concentrations of ADMA (**a**) and SDMA (**b**) in the initial blood sample of hospitalized COVID-19 patients (N = 31). Boxes show median ± interquartile range, with whiskers representing 10th to 90th percentile; outliers are plotted individually.

**Figure 2:**
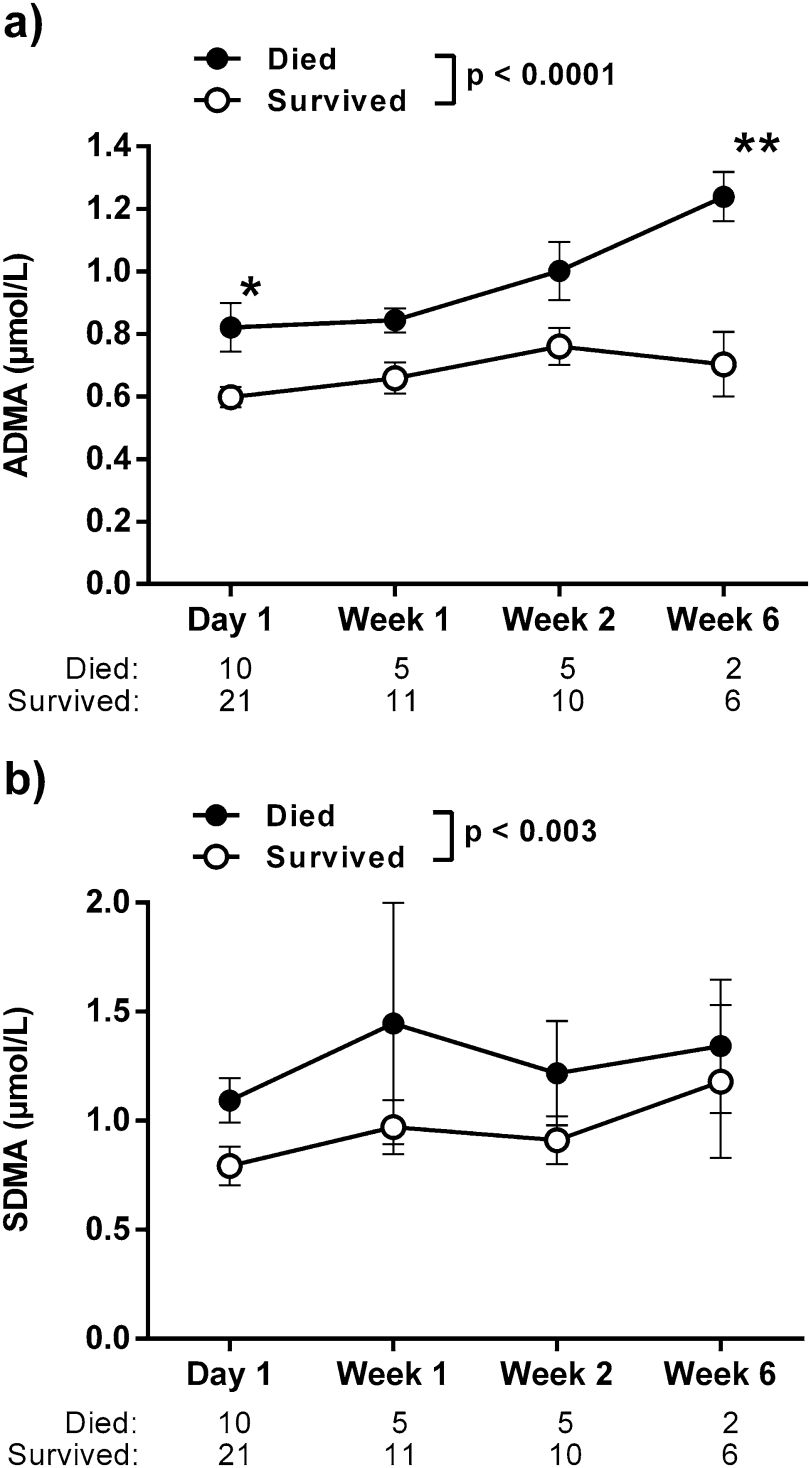
Time course of ADMA (**a**) and SDMA (**b**) serum concentrations of hospitalized COVID-19 patients during in-hospital treatment. N refers to the number of patients for whom blood samples were available for analysis at each time point. *P* values given in the legend refer to two-sided ANOVA for trend between groups; asterisk mark statistically significant differences between both group at a specific time point in Bonferroni’s multiple comparisons test. *p < 0.05, **p < 0.01 between groups.

The serum concentrations of ADMA and SDMA at hospital admission were significantly higher in patients who experienced in-hospital death versus those who survived (ADMA, 0.86 ± 0.07 µmol/L *vs.* 0.59 ± 0.03 µmol/L, *p* = 0.0004; SDMA, 1.15 ± 0.09 µmol/L *vs.* 0.78 ± 0.08 µmol/L, *p* = 0.017; Fig. 1). The ADMA serum concentration further increased significantly over time in patients who died, but not in survivors (Fig. 2a). The difference of SDMA levels between both groups remained significant during the course of hospitalization (Fig. 2b). The differences in ADMA and SDMA between survivors and non-survivors were stable when six patients with pre-existing chronic kidney disease were excluded (supplementary Fig. S3); the same was true for the comparison of eight patients admitted with ongoing mechanical ventilation versus those who were not (supplementary Fig. S4).

### Association of biomarkers with COVID-19-related outcome

ADMA and SDMA were differentially associated with organ injury evolving during COVID-19: Both ADMA and SDMA were significantly higher in 14 patients who developed cardiac injury (ADMA, 0.79 ± 0.06 vs. 0.57 ± 0.04 µmol/L, *p* = 0.048; SDMA, 1.02 ± 0.08 vs. 0.78 ± 0.11 µmol/L, *p* = 0.04). ADMA was also higher in 14 patients with ARDS (0.78 ± 0.07 vs. 0.58 ± 0.03 µmol/L, *p* = 0.01) and tended to be higher in 11 patients with liver injury, but this trend did not reach statistical significance. SDMA was significantly higher in patients with liver injury (1.07 ± 0.16 vs. 0.79 ± 0.07 µmol/L, *p* = 0.017) and showed a non-significant trend towards higher levels in ARDS. None of the two biomarkers was different in patients with (N = 8) or without acute kidney injury. The differences in the two biomarkers in these diseases are graphically shown in supplementary Fig. S5.

We performed ROC analyses with mortality as outcome for SDMA, ADMA, SOFA score, and other blood biomarkers that were significantly elevated at admission in non-survivors. The SOFA score showed an AUC of 0.819 (95% CI, 0.659–0.980; cut-off, 5.5 points; *p* = 0.007); CRP had an AUC of 0.843 (95% CI, 0.702–0.985; cut-off, 94.1; *p* = 0.003), and PCT had an AUC of 0.876 (95% CI, 0.745–1.000; cut-off, 0.32; *p* = 0.001). Leukocyte cell count had an AUC of 0.833 (95% CI, 0.687–0.980; cut-off, 10.7; *p* = 0.004). The ROC curves for these inflammatory parameters are given in supplementary Fig. S6.

**Figure 3:**
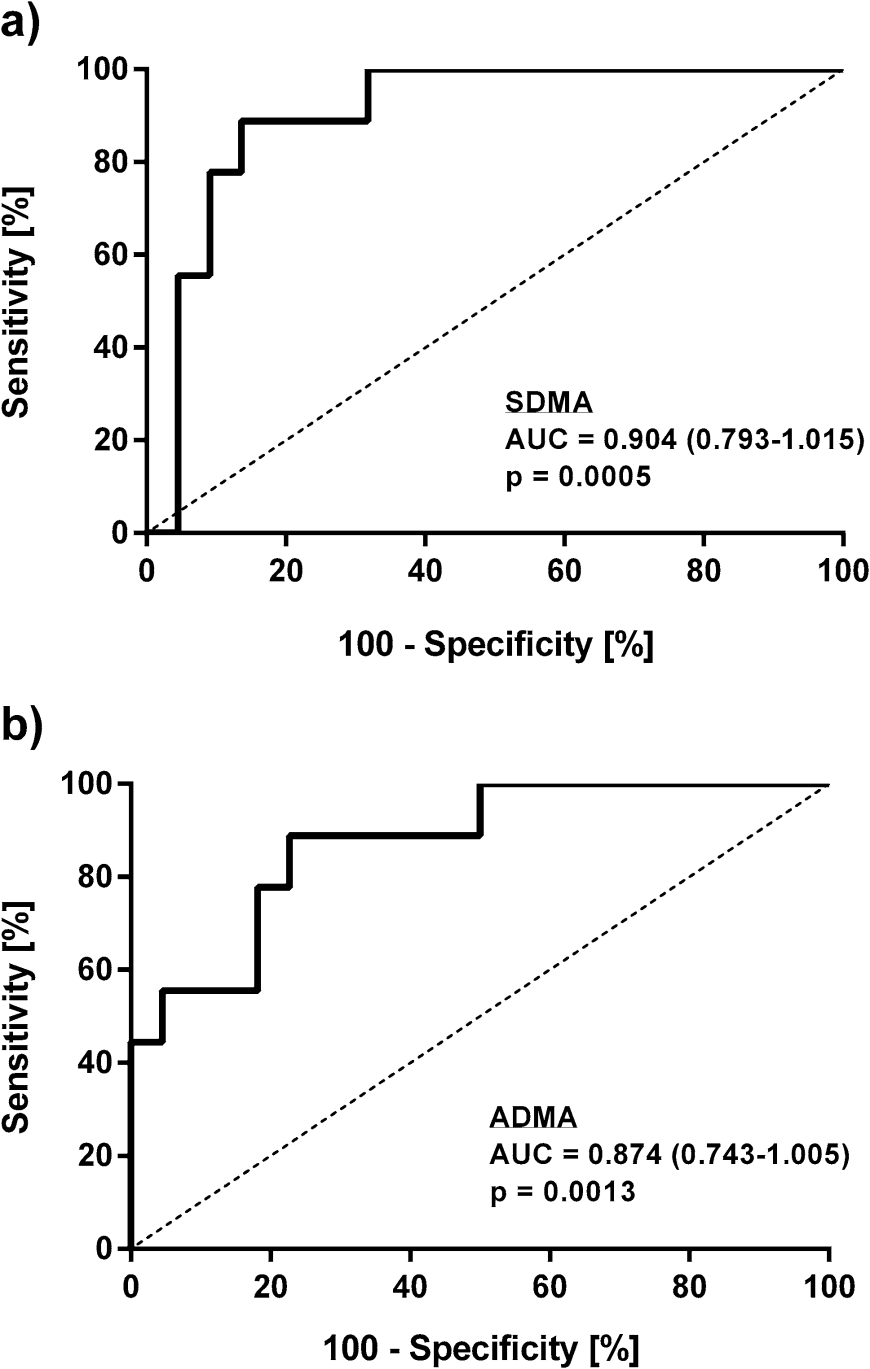
Receiver-operated curve (ROC) analyses of ADMA (**a**) and SDMA (**b**) for in-hospital mortality. AUC, area under the curve.

ROC analysis further showed that an ADMA concentration of 0.66 µmol/L allowed to discriminate survivors from patients who experienced in-hospital death with 88.9% sensitivity and 81.1% specificity (AUC, 0.874 (95% CI, 0.743–1.000), *p* = 0.0013; Fig. 3a). The cut-off for SDMA was 0.90 µmol/L (sensitivity, 88.9%, specificity, 89.2%; AUC, 0.904 (95% CI, 0.793–1.000); *p* = 0.0005; Fig. 3b).

### Prediction of COVID-19 mortality

The HR of in-hospital death for patients with a SOFA score ≥ or < 5.5 was 1.25 (95% CI, 0.29–5.66; *p* = n.s.). Addition of SDMA to the survival model significantly improved prediction of mortality; the HR was 6.31 for patients with high SOFA and high SDMA vs. those with both values low (95% CI, 1.07–37.16; *p* = 0.04). Likewise, neither CRP nor PCT when analyzed alone were significantly predictive of in-hospital death. For both inflammatory markers, addition of SDMA significantly improved predictive power (supplementary Fig. S7 and Table 2).

**Table 2:** Predictive power of the SOFA score, C-reactive protein levels, and pro-calcitonin levels at admission for in-hospital mortality, when analysed alone or in combination with SDMA or ADMA.

Parameter	Mortality [%] high/intermediate^1^/low	P*	Hazard Ratio for high vs. low group	*p*^#^
SOFA Score	50.0/15.4	n.a	1.25 (0.29–5.66)	0.760
SOFA Score + SDMA	75.0/33.3/0.0	0.020	6.31 (1.07–37.16)	0.042
SOFA Score + ADMA	77.8/11.1/9.1	0.123	2.38 (0.46–11.19)	0.528
C-reactive protein	57.1/5.9	n.a	3.47 (0.70–12.01)	0.176
C-reactive protein + SDMA	77.8/28.6/0.0	0.005	7.95 (1.57–40.25)	0.0012
C-reactive protein + ADMA	77.8/22.2/0.0	0.039	5.04 (0.81–31.28)	0.083
Pro-calcitonin	61.5/5.6	n.a	3.54 (0.84–14.90)	0.085
Pro-calcitonin + SDMA	87.5/25.0/0.0	0.004	10.37 (2.02–53.35)	0.005
Pro-calcitonin + ADMA	87.5/20.0/0.0	0.017	5.58 (0.93–33.53)	0.060
Leukocytes	58.3/10.5	n.a	4.71 (1.15–16.09)	0.032
Leukocytes + SDMA	100.0/27.3/0.0	< 0.0001	25.97 (4.49–150.30)	0.0003
Leukocytes + ADMA	85.7/27.3/0.0	0.012	8.30 (1.42–48.38)	0.019

^1^ Mortality of the subgroups is given for the dichotomized SOFA score, C-reactive protein, and pro-calcitonin values when analyzed alone (no intermediate risk group), as well as for the highest risk group (traditional risk marker high plus SDMA/ADMA high), the intermediate risk group (traditional risk marker or SDMA/ADMA high), and lowest risk group (traditional risk marker plus SDMA/ADMA low). *P denotes statistical significance level in logrank test for trend across all risk groups. ^#^*p* denotes statistical significance level for the hazard ratio of the high vs. low-risk groups. SOFA, sequential organ failure assessment; SDMA, symmetric dimethylarginine; ADMA, asymmetric dimethylarginine.

**Figure 4:**
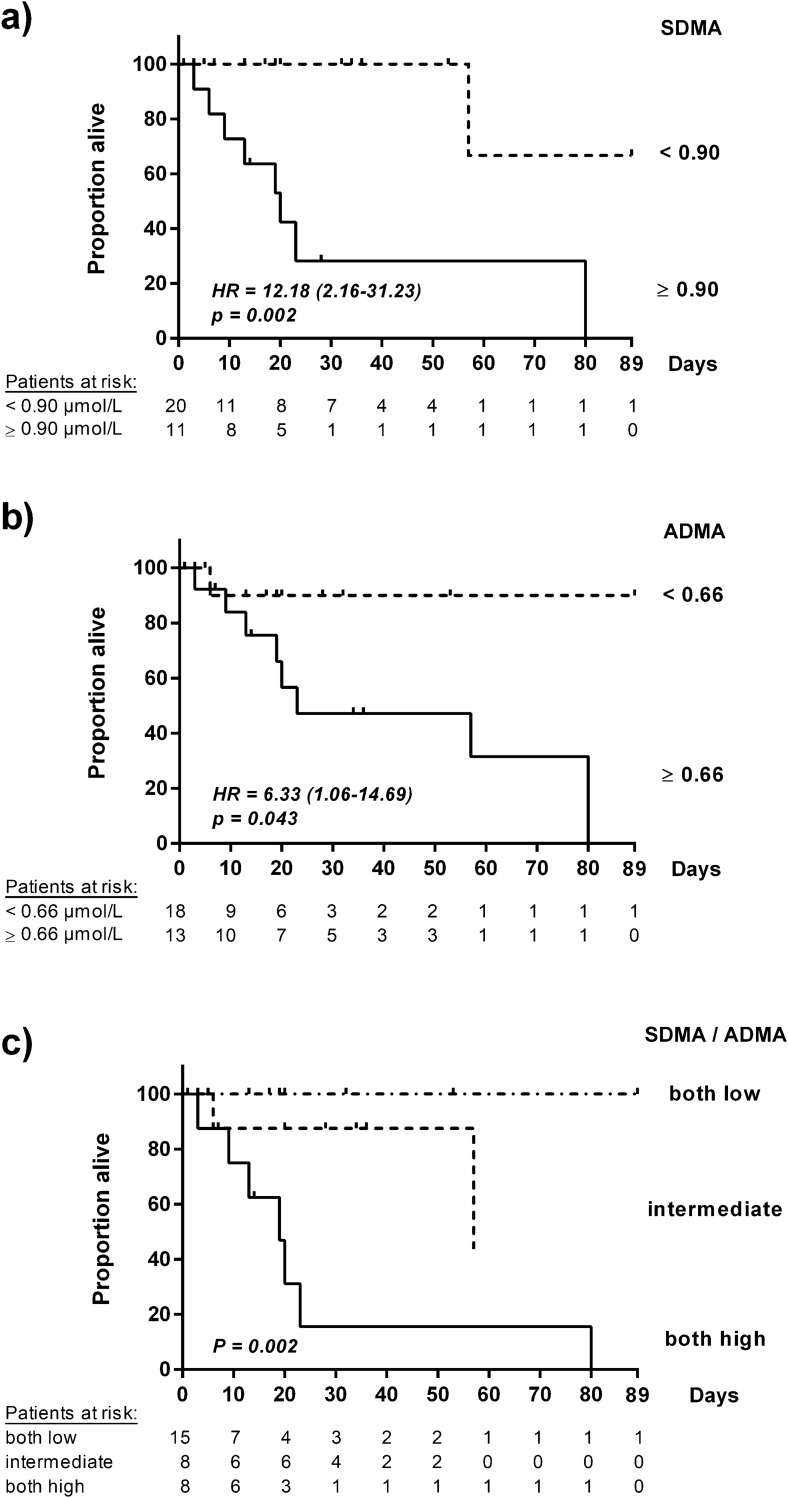
Kaplan–Meier curves for in-hospital mortality of hospitalized COVID-19 patients stratified for biomarker concentrations (N = 31): SDMA (**a**), ADMA (**b**), sequential quantification of SDMA and ADMA (**c**). The x-axis displays days after hospital admission.

COVID-19 patients with ADMA serum concentration ≥ 0.66 µmol/L had a significantly higher probability of in-hospital death than those with ADMA < 0.66 µmol/L. The HR was 6.33 (95% CI, 1.06–14.69), *p* = 0.043 (Fig. 4a). Individuals with SDMA serum concentration ≥ 0.90 µmol/L had a HR for in-hospital death of 12.18 (95% CI, 2.16–31.23), *p* = 0.002 (Fig. 4b).

We performed multivariable-adjusted logistic regression analyses with SDMA and ADMA above and below the cut-off concentrations as categorical variables. Both markers were significantly associated with in-hospital mortality in models adjusted for age and sex and for age, sex, and eGFR (Table 3). In a fully adjusted model including inflammatory markers, SDMA remained highly significantly associated with survival, whilst this association lost significance for ADMA (Table 3).

**Table 3:** Stepwise multivariable-adjusted regression analysis for SDMA and ADMA with in-hospital mortality.

	SDMA		ADMA	
	HR	*P*	HR	*p*
Model 1^a^	62.5 (4.0–1,000.0)	0.003*	34.2 (2.3–500.4)	0.010*
Model 2 ^b^	166.7 (4.0–1,000.0)	0.007*	31.9 (2.3–445.9)	0.010*
Model 3 ^c^	333.3 (1.2–1,000.0)	0.044*	10.3 (0.6–190.5)	0.118

^a^Model 1 was adjusted for age and sex. ^b^Model 2 was adjusted for age, sex, and eGFR. ^c^Model 3 was adjusted for age, sex, eGFR, C-reactive protein, and pro-calcitonin. *denotes statistically significant associations with in-hospital mortality.

We next tested whether SDMA and ADMA combined in a single variable improved predictive power. However, neither (SDMA + ADMA) nor (SDMAxADMA) showed significant improvement over SDMA used alone (supplementary Fig. S8). In addition, we tested three previously published COVID-19 mortality risk scores ^5,7,8^; none of these scores showed a significant prediction of mortality in our patient cohort (supplementary Fig. S9).

**Figure 5:**
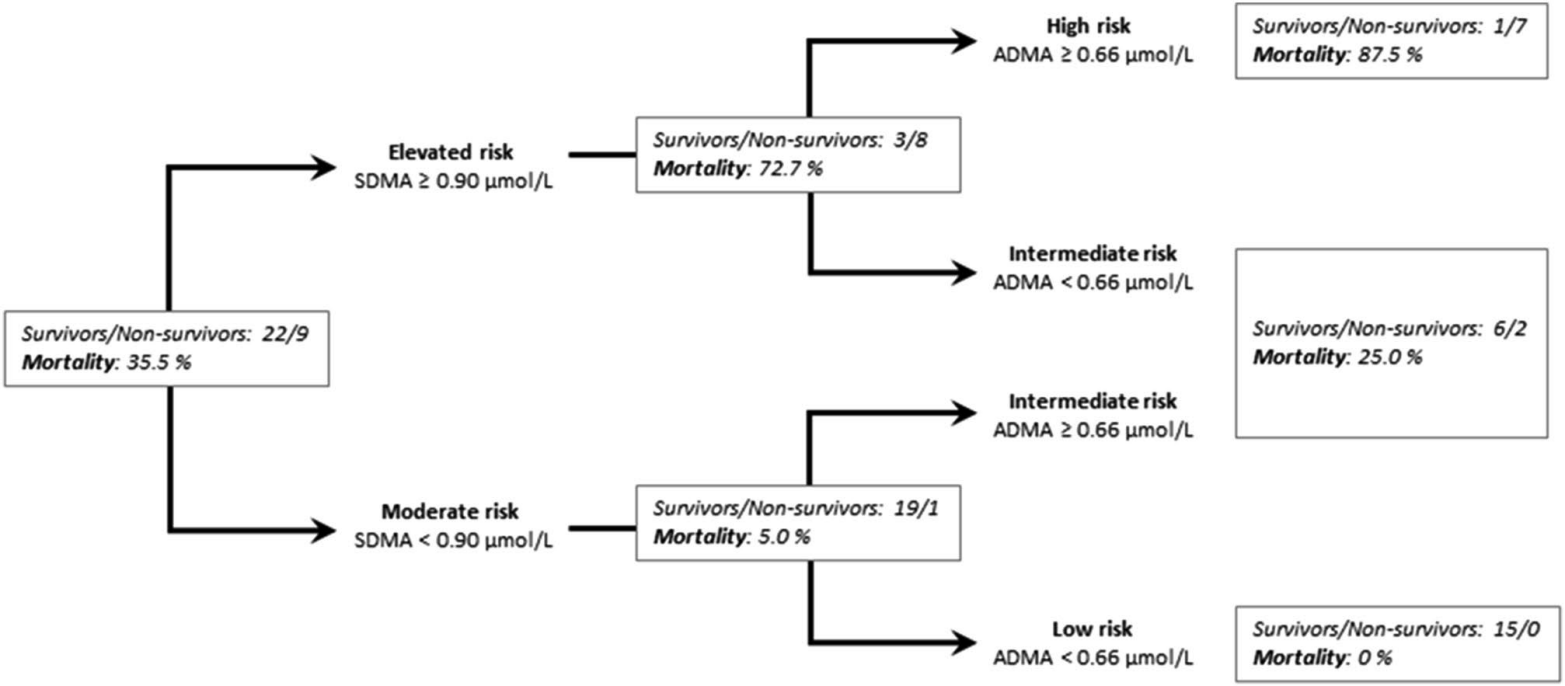
Decision tree analysis to identify the risk of in-hospital mortality. Out of 31 patients of whom serum samples were available for day 1, nine died (30%). First decision step: Patients were identified as having elevated risk when SDMA levels were ≥ 0.90 μmol/L, and moderate risk when SDMA levels were < 0.90 µmol/L. Second decision step: Additional analysis of ADMA allowed identification of patients with high risk (SDMA ≥ 0.90 µmol/L and ADMA ≥ 0.66 µmol/L (mortality, 87.5%), intermediate risk (SDMA ≥ 0.90 µmol/L or ADMA ≥ 0.66 µmol/L (mortality, 25%), or low risk (SDMA < 0.90 µmol/L and ADMA < 0.66 µmol/L (mortality, 0%).

By contrast, we observed that sequential measurements of SDMA and ADMA significantly enhanced discrimination of mortality risk. Patients with high SDMA and high ADMA concentrations had a HR of in-hospital mortality of 9.30 (95% CI, 2.09–41.37), *p* = 0.0034, as compared to those with both biomarker levels low; individuals with only one biomarker level elevated had an intermediate risk (*p* = n.s. vs. both biomarkers low; Fig. 4c). Using a decision tree algorithm, we were able to discriminate high-risk patients (SDMA ≥ 0.90 µmol/L and ADMA ≥ 0.66 µmol/L) with an in-hospital mortality of 87.5%, intermediate-risk patients (either SDMA or ADMA elevated) with an in-hospital mortality of 25%, and low-risk patients (SDMA < 0.90 µmol/L and ADMA < 0.66 µmol/L), whose in-hospital mortality was 0% (Fig. 5). Sequential measurement of SDMA and ADMA therefore provided the best predictive power for in-hospital death when compared to traditional risk markers or their combination with either SDMA or ADMA.

## Discussion

The present study is the first to report two novel biomarkers beyond currently established clinical chemistry and blood hematology parameters to identify hospitalized COVID-19 patients at high risk of in-hospital mortality. These biomarkers, SDMA and ADMA, have a high sensitivity and specificity to predict mortality amongst hospitalized COVID-19 patients.

In the general population, pre-existing conditions like advanced age, obesity, type 2 diabetes, and hypertension are risk factors for a severe course of COVID-19 requiring hospitalization^30,31^. In our cohort of hospitalized COVID-19 patients, however, none of these conditions was significantly associated with in-hospital mortality or organ dysfunction. This is not astonishing, as 30 out of the 31 patients had pre-existing conditions and the mean age of our cohort was above 60 years. Although severe COVID-19 is an inflammation-driven disease, leukocyte cell count was the only traditional inflammatory marker that was weakly associated with mortality, whilst neither the SOFA score nor other commonly used laboratory parameters like CRP and PCT were able to significantly identify high-risk patients. In addition, three previously published risk scores that are based on a variety of different traditional diagnostic parameters^5,7,8^ failed to significantly predict mortality in our COVID-19 cohort.

Current data suggest that proper function of endothelial NO synthase (eNOS) may be an important mechanism of defense after infection with SARS-CoV-2^32^. Endothelial cell tropism of the SARS-CoV-2 virus causes endothelial inflammation^14^, which possibly accelerates endothelial dysfunction and NO deficiency^33^. Endothelial dysfunction as marked by dysfunctional endothelium-dependent, NO-mediated vasodilation, and the ensuing high thrombosis risk contribute to COVID-19 morbidity and mortality^34^. ADMA is an endogenous, competitive inhibitor of NO synthesis an—like its congener molecule, SDMA—an inhibitor of cellular L-arginine uptake^18^. Both dimethylarginines are formed through the action of protein arginine methyltransferases (PRMTs), enzymes that are involved in innate immune responses and in the response to hypoxia in the lung^19^. PRMTs produce mainly SDMA in the central nervous system, but ADMA in many other organs including heart, circulatory system, and lungs (for review, cf.^17^). ADMA is enzymatically degraded by dimethylarginine dimethylaminohydrolases (DDAH), the activity of which is reduced by cysteine nitrosylation^35^, resulting in ADMA accumulation in a manner reversible by antioxidants. SDMA, by contrast, is inactivated by alanine-glyoxylate aminotransferase (AGXT2), an enzyme predominantly expressed in the kidneys and liver^36^. The underlying biochemistry may explain the associations of ADMA and SDMA with ensuing organ dysfunction typical for COVID-19 patients, i.e. acute kidney injury, cardiovascular thromboembolic events, neurological damage, and multi-organ failure. ADMA and SDMA, two biomarkers causing impaired NO production, may interfere with two essential pathophysiological steps in COVID-19-associated critical illness: vascular failure and immune response. Whilst the interaction of ADMA and SDMA with endothelial NO production has been extensively studied, there is much less information available on their interaction with inducible NO synthase^20^. Nonetheless, both mechanisms may contribute to their roles as predictive biomarkers in the present cohort. In line with this, we have previously reported that sequential measurement of SDMA and ADMA helps to predict the lethality of sepsis in ICU-treated patients^26^. Others have also reported ADMA to be associated with ICU death in a heterogeneous cohort of critically ill patients^27^.

Respiratory failure and global hypoxemia were major pathophysiological problems that led to hospital admission of the patients included in our present study. We have previously shown that ADMA continuously increases in humans exposed to chronic-intermittent hypoxia^37^, and that DDAH1^-/-^ mice that have high circulating ADMA concentration are prone to develop pulmonary hypertension and right ventricular hypertrophy upon exposure to chronic hypoxia^38^. Based upon our data showing that inhibitors of NO synthesis are predictive biomarkers for COVID-19 survival with high discriminative power, and in line with published pilot studies that successfully administered inhaled NO to treat severe respiratory failure^39,40^, therapeutic approaches aimed at restoring physiological NO function may help to better treat severe COVID-19.

Combined analysis of ADMA and SDMA was previously shown by us to predict mortality risk in sepsis patients^26^. In that study, critically ill patients during ICU treatment were included, the cut-off values used were 1.34 µmol/L for SDMA and 0.97 µmol/L for ADMA. In the present cohort of COVID-19 patients, we found lower cut-off values for both biomarkers, which is in line with the unselected character of the present cohort, including hospitalized COVID-19 patients with a moderate to severe disease course and 19 out of a total of 31 patients being treated on ICU.

This is a retrospective cohort study and therefore has certain limitations. The study was carried out at a single center and involved a relatively small number of patients, which did not allow us to perform extensive subgroup analyses. The population comprised exclusively hospitalized patients with confirmed COVID-19 in a tertiary care hospital, with a relevant portion of patients that were referred as secondary admissions from other hospitals in the region, mostly but not exclusively because of ARDS. Although we used a validated liquid chromatography-tandem mass spectrometric (LC–MS/MS) method for biomarker analysis, ADMA and SDMA may be measured by ubiquitously available laboratory methods. We have previously validated an ADMA enzyme-linked immunosorbent assay (ELISA) and established reference ranges using this method^41^. However, in the absence of a widely used routine analytical method for ADMA and SDMA, reference ranges reported in the literature are broad and may vary according to the analytical method applied, and cut-off values reported in this study relate to the analytical method we used, LC–MS/MS. Nevertheless, our observations warrant follow-up studies with larger patient groups and a more formalized statistical approach to confirm the utility of SDMA and ADMA to independently predict COVID-19 outcome and severity.

In conclusion, we show here for the first time that ADMA and SDMA are biomarkers that allow us to prospectively identify COVID-19 patients with a high mortality risk beyond the diagnostic utility of the SOFA score and commonly used laboratory parameters. This may help to monitor such patients more closely, establish intensive care treatment earlier, and reduce the lethality of the COVID-19 pandemic.

## Patients and methods

### Study cohort and protocol

31 consecutive patients with confirmed SARS-CoV-2 infection were primarily admitted with symptomatic COVID-19 to the University Hospital Aachen (UKA) or referred from another hospital between March and May 2020. Patients were included in this study if the main cause for hospital admission was COVID-19 disease. Patients had to have a positive SARS-CoV-2 test result in respiratory samples that was performed in our hospital or externally before admission. Patients were only included if an initial blood sample for biomarker analysis was available within 24 h after admission. All patients gave their informed consent to have their blood samples included into the RWTH centralized Biomaterial Bank (RWTH cBMB) for further scientific study. The Ethics Committee of the Medical Faculty of RWTH Aachen had consented the Covid-19 Aachen study (COVAS) according to their vote EK080/20 and the regulations of the RWTH CBMB (vote EK206/09). All investigations were performed in accordance with the Declaration of Helsinki in its latest revision. No further selection criteria were applied.

All patients were treated according to best medical practice and individual clinical needs. Patients were either isolated under standard care or treated in our intensive care unit (ICU). The decision on treatment strategies was based on clinical judgment of the severity of the disease and the presence or absence of acute respiratory distress syndrome (ARDS). Severity of ARDS was classified according to the degree of hypoxia as defined by the “Berlin definition”^42^.

Comorbidities (such as hypertension, overweight or obesity, diabetes, pre-existing respiratory or cardiovascular diseases, smoking, chronic kidney disease, malignancies, chronic liver disease), and medications prescribed at the time of admission were recorded in hospital, or taken from existing medical records. Blood samples were drawn into EDTA vacutainers on day 1 after admission, after one week, two weeks, and six weeks. Samples were immediately centrifuged and stored at -80 °C until analysis. Sample storage and logistics were managed by the team of the RWTH cBMB.

### Measurement of ADMA and SDMA by LC–MS/MS

Validated protocols for liquid chromatography-tandem mass spectrometry (LC–MS/MS) were used to quantify ADMA and SDMA in serum^43^. Briefly, 25 μl of serum were diluted in methanol to which stable isotope labelled internal standards had been added. Subsequently, the compounds were converted into their butyl ester derivatives and quantified by LC–MS/MS (Xevo TQ-S cronos, Waters GmbH, Eschborn, Germany). Compounds were separated on an Aquity UPLC BEH C18 column (2·1 × 50 mm, 1.7 µm, Waters GmbH). The coefficient of variation for the quality control samples was below 15% for both compounds.

### Clinical and biochemical assessment of patient status

Patients were assessed for eligibility based on a positive RT-PCR assay for SARS-CoV-2 in a respiratory tract sample as previously described^30,44^. Vital parameters presented in this study were taken between four and 24 h following hospital admission or intubation, with the worst values being depicted. We defined multi organ failure (MOF) as a failure of at least four major organs (heart, lungs, liver, kidneys) because of complications of COVID 19. Severity of ARDS was defined using P/F-ratio or the Horowitz index. Acute kidney injury was defined according to the AKIN criteria^45^ and/or need for continuous veno-venous hemofiltration in patients with no pre-existing chronic renal failure. Cardiac injury was defined as troponin T levels > 52 ng/mL or a relative increase by ≥ twofold during in-hospital treatment. Liver injury was defined as an increase in serum total bilirubin by ≥ twofold and/or increases in serum ALT and/or AST activities by ≥ threefold. High thromboembolic burden was defined as a relative increase of D-dimer levels by ≥ twofold during in-hospital treatment. Circulatory insufficiency/shock was defined as the need for catecholamines at any time during in-hospital treatment. Febrile days were defined as the time from fever onset until the last documented value above 38.5 ℃. Patients with a body mass index (BMI) of 25 to < 30 kg/m^2^ were classified as overweight and those with BMI ≥ 30 kg/m^2^ as obese. Diabetes and prediabetes were defined by clinical history, medication and HbA_1c_ values ≥ 6.5% or ≥ 5.7 to < 6.5%, respectively. Serum and whole blood samples were obtained routinely at the time of admission. Complete blood count, coagulation tests, inflammatory markers [circulating levels of C-reactive protein (CRP), pro-calcitonin (PCT)] and creatinine levels in blood were measured among other tests. Creatinine clearance was estimated using the CKD-EPI formula^46^.

### Statistical analyses

All variables were tested for normal distribution using the Kolmogorov–Smirnov test. Data are presented as mean with standard deviation (SD). Differences between groups were tested for significance using the nonparametric Mann–Whitney U test for two groups or the Kruskal–Wallis analysis of variance for more than two groups. The Chi^2^ test was used for comparison of categorical variables between groups. Time courses of ADMA and SDMA concentrations were examined using repeated measures two-way ANOVA followed by Tukey’s multiple comparisons test. Spearman’s rank correlation was used to assess pairwise correlations. Survival analyses were performed using Kaplan–Meier curves comparing patients with ADMA and SDMA above or below the cut-off value determined in receiver-operated curve (ROC) analyses. Hazard ratios (HR) and 95% confidence intervals (CI) were calculated by multivariable-adjusted logistic regression analyses. As we identified two biomarkers, ADMA and SDMA, as predictors of COVID-19 mortality, we analyzed additional models using (SDMA + ADMA) or (SDMAxADMA) as variables, respectively. In addition, we performed a decision tree analysis to determine risk upon sequential analysis of SDMA and ADMA. Cut-offs to separate risk groups were based on values determined in ROC analysis for both biomarkers. All statistical analyses were performed using SPSS (version 25; IBM Corporation, Armonk, NY, USA) and GraphPad Prism (version 6.01, GraphPad Software, San Diego, CA, USA). For all tests, p < 0.05 was considered statistically significant.

## Supplementary information

Supplementary Figures.

## Data Availability

The datasets generated during and/or analyzed during the current study are available from the corresponding author on reasonable request.
