# Supplementary material for: Elevated serum SDMA and ADMA at hospital admission predict in-hospital mortality of COVID-19 patients
Source: Sci Rep. 2021 May 10;11:9895. doi: 10.1038/s41598-021-89180-w (PMC8110746; doi:10.1038/s41598-021-89180-w)
Supplement: Supplementary file 1 — Supplementary Figures. [file 41598_2021_89180_MOESM1_ESM.pdf]

Elevated serum SDMA and ADMA at hospital admission predict in-hospital mortality of COVID-19 patients

Juliane Hannemann, Paul Balfanz, Edzard Schwedhelm, Bojan Hartmann, Johanna Ule,
Dirk Müller-Wieland, Edgar Dahl, Michael Dreher, Nikolaus Marx, Rainer Böger

Supplementary Figures

Supplementary Figure S1: Correlations of ADMA.

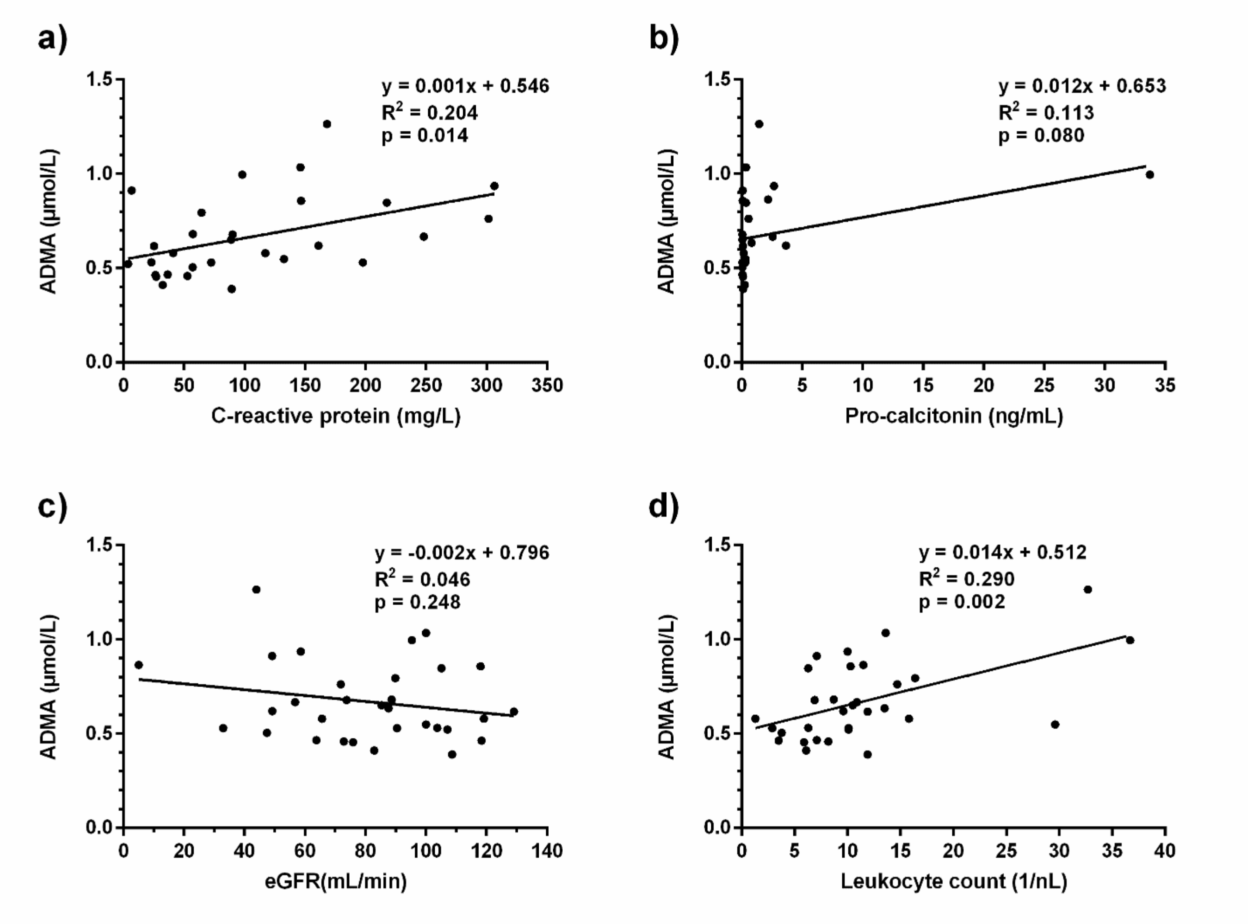

Legend: Data show correlations of ADMA concentration on the day of hospital admission with C-reactive protein (a), pro-calcitonin (b), estimated glomerular filtration rate (eGFR, c), and leukocyte cell count (d), all assessed on the same day.

Supplementary Figure S2: Correlations of SDMA.

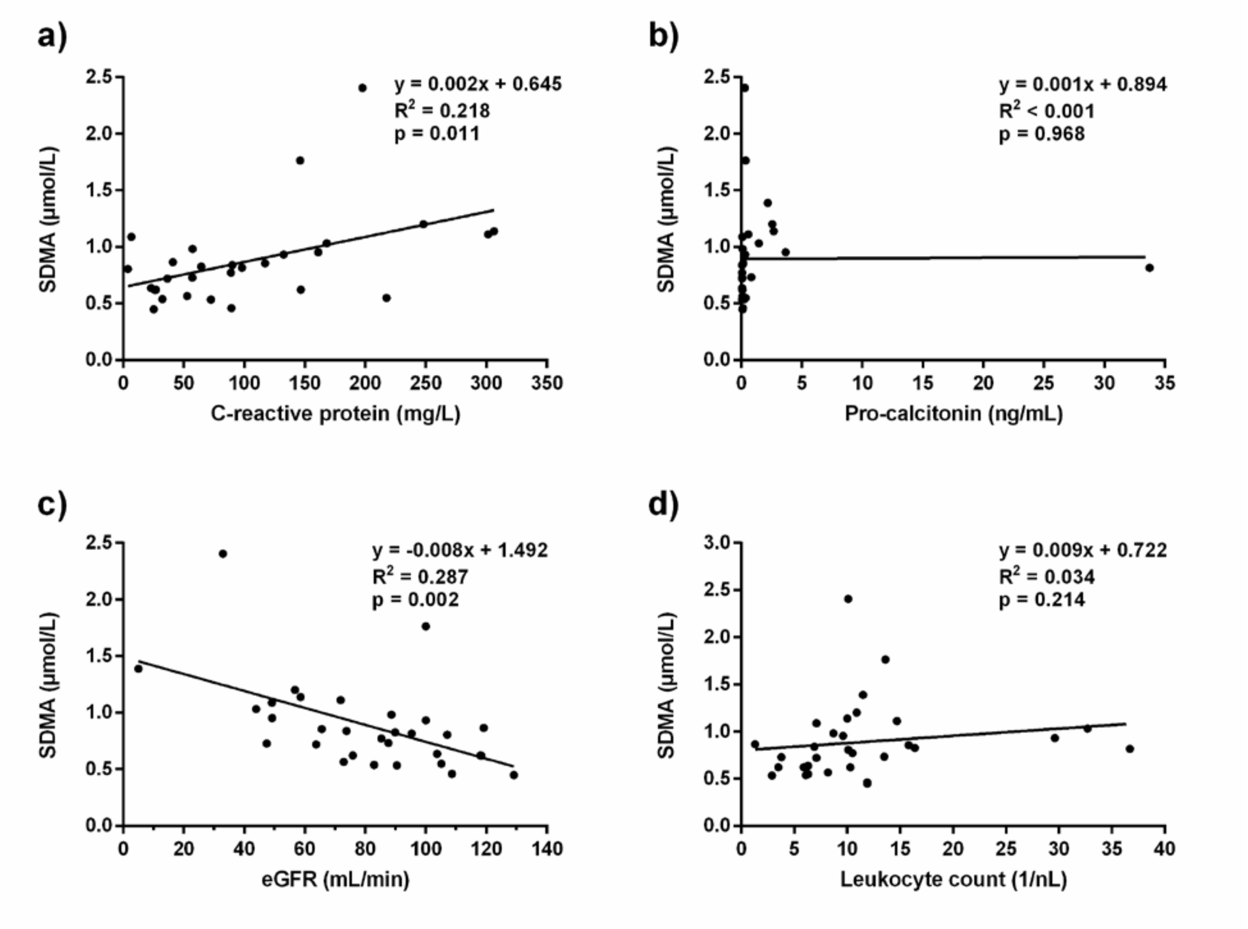

Legend: Data show correlations of SDMA concentration on the day of hospital admission with C-reactive protein (a), pro-calcitonin (b), estimated glomerular filtration rate (eGFR, c), and leukocyte cell count (d), all assessed on the same day.

Supplementary Figure S3: ADMA and SDMA in COVID-19 patients without pre-existing chronic kidney disease.

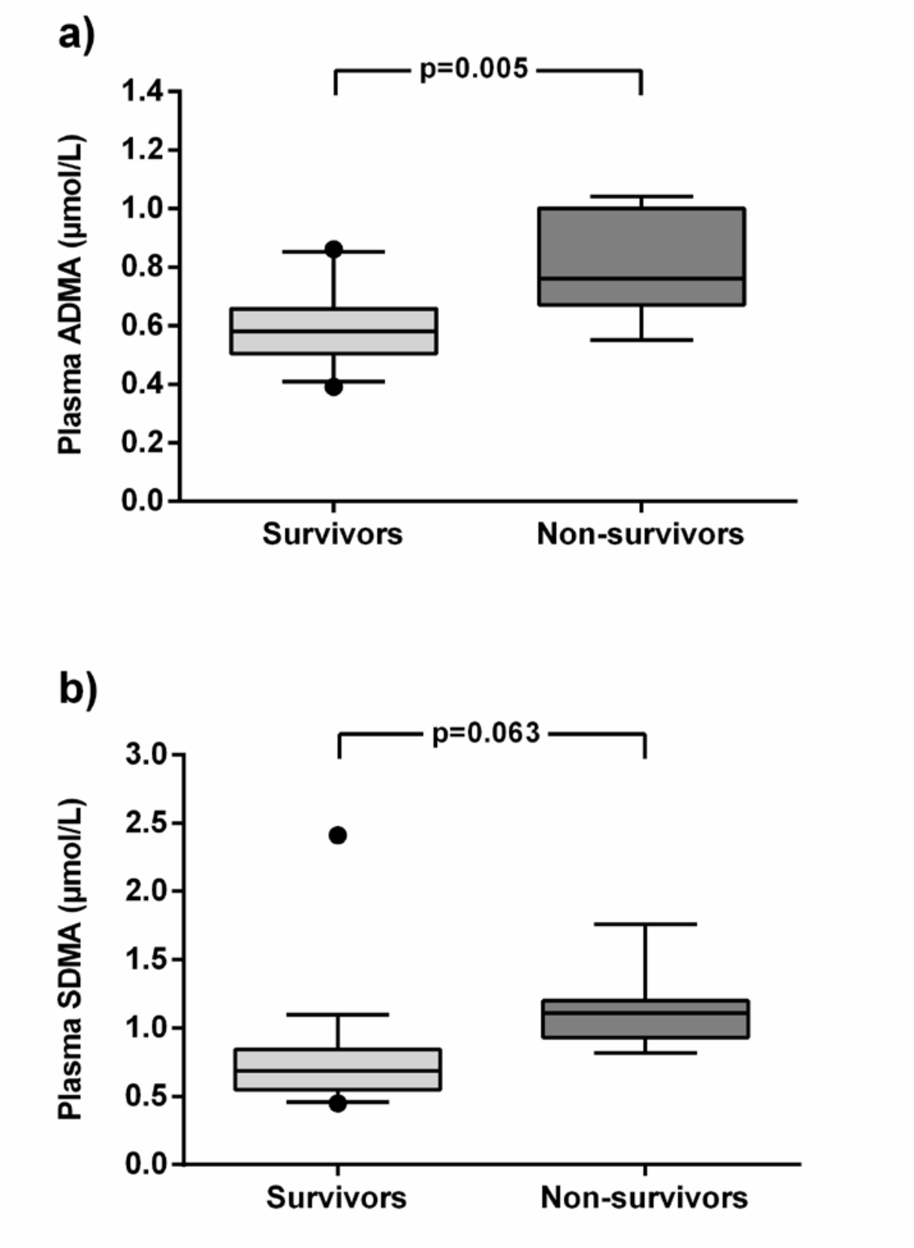

Legend: Box plots show serum concentrations of ADMA (a) and SDMA (b) on the day of hospital admission of COVID-19 patients without pre-existing chronic kidney disease (N = 25). Boxes show median ± interquartile range, with whiskers representing 10th to 90th percentile; outliers are plotted individually.

Supplementary Figure S4: ADMA and SDMA in COVID-19 patients with or without ongoing mechanical ventilation at hospital admission.

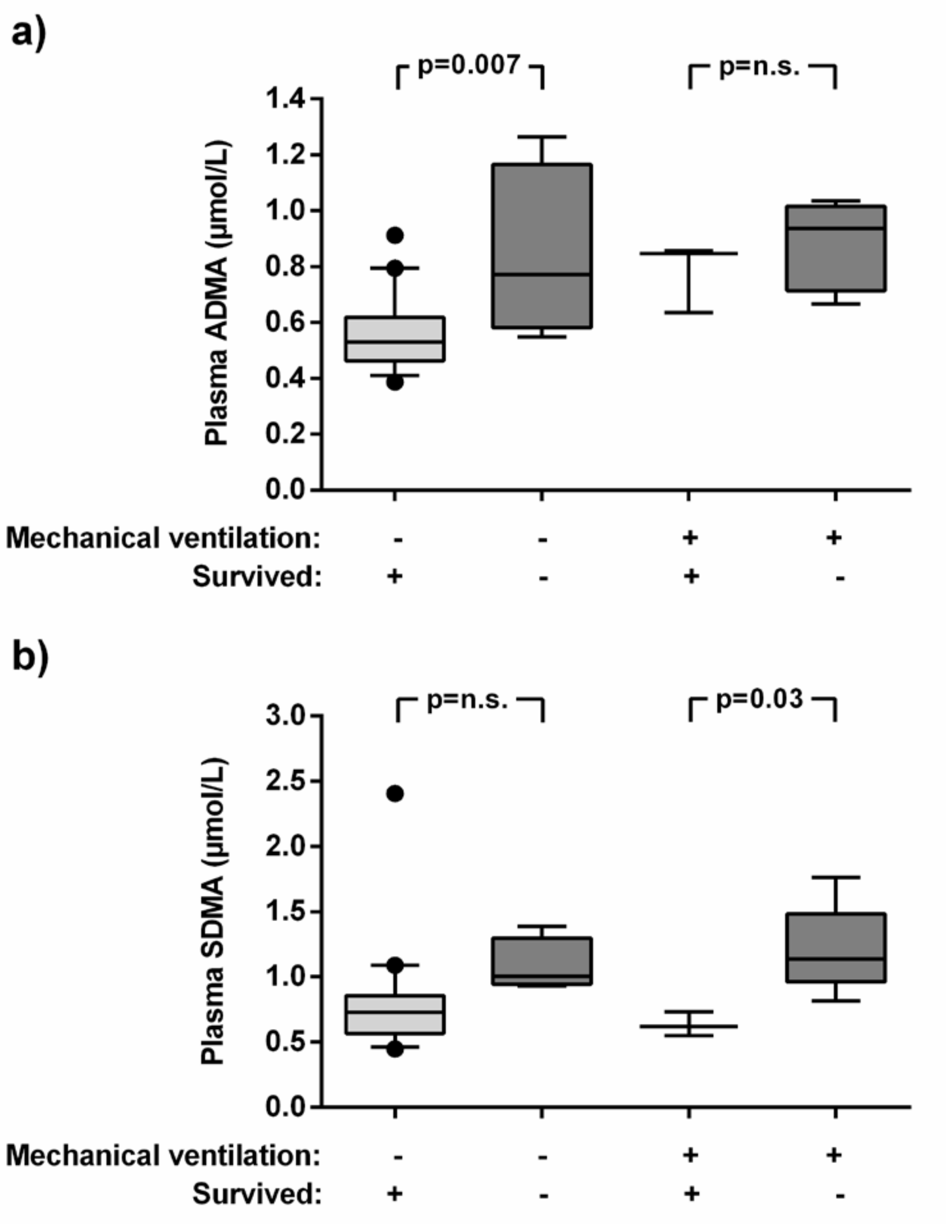

Legend: Box plots show serum concentrations of ADMA (a) and SDMA (b) on the day of hospital admission of COVID-19 patients who were admitted with ongoing mechanical ventilation (N = 8) or not (N = 23). Boxes show median ± interquartile range, with whiskers representing 10th to 90th percentile; outliers are plotted individually.

Supplementary Figure S5: ADMA and SDMA concentrations in COVID-19 patients who developed organ injury during in-hospital treatment as compared to those who did not.

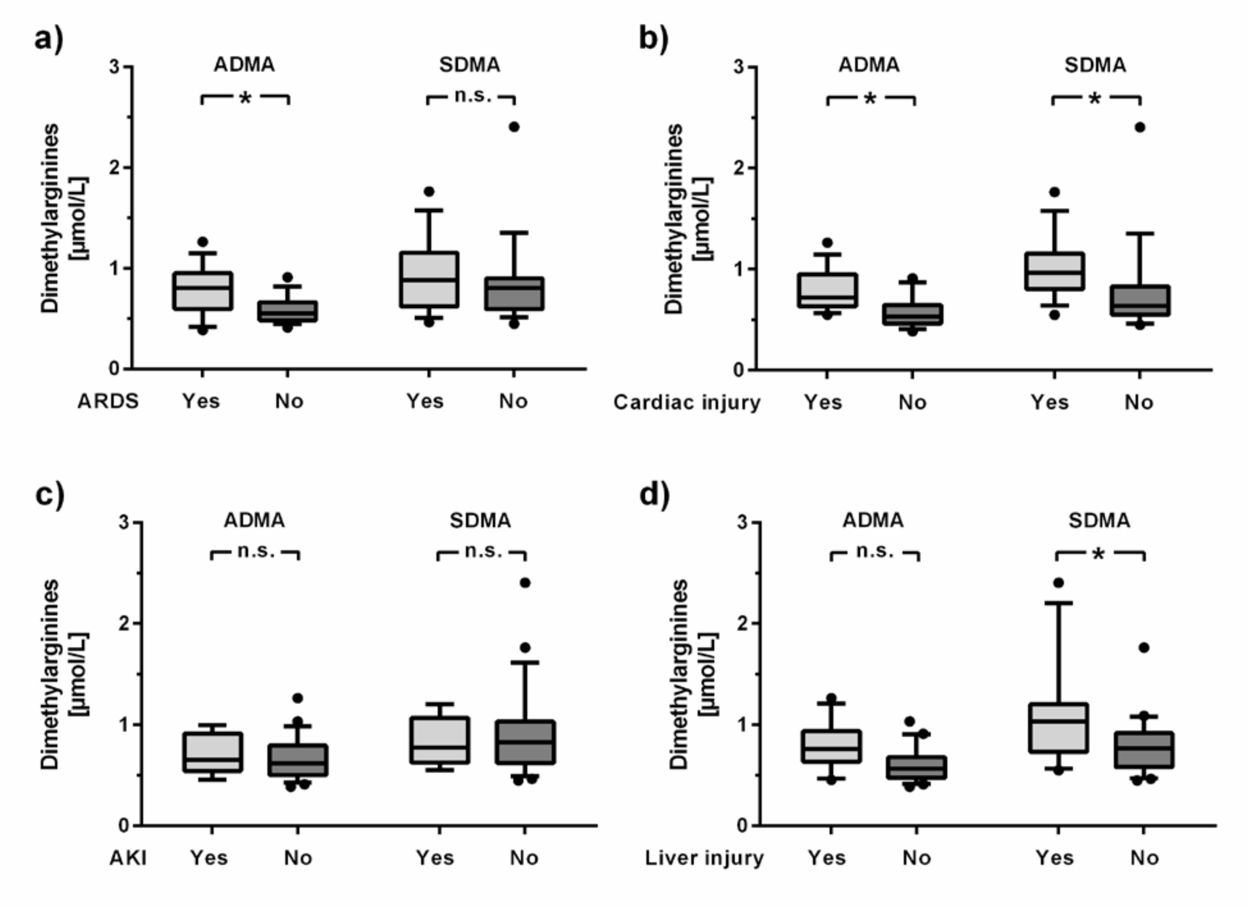

Legend: Box plots show serum concentrations of ADMA and SDMA on the day of hospital admission of COVID-19 patients who developed COVID-19-associated organ injury during the course of in-patient treatment: a) acute respiratory distress syndrome (ARDS), b) cardiac injury, c) acute kidney injury (AKI), d) liver injury. Organ injuries were defined as described in the Methods section. Boxes show median ± interquartile range, with whiskers representing 10th to 90th percentile; outliers are plotted individually.

Supplementary Figure S6: Receiver-operated curve (ROC) analyses of SOFA score and inflammatory markers for in-hospital mortality.

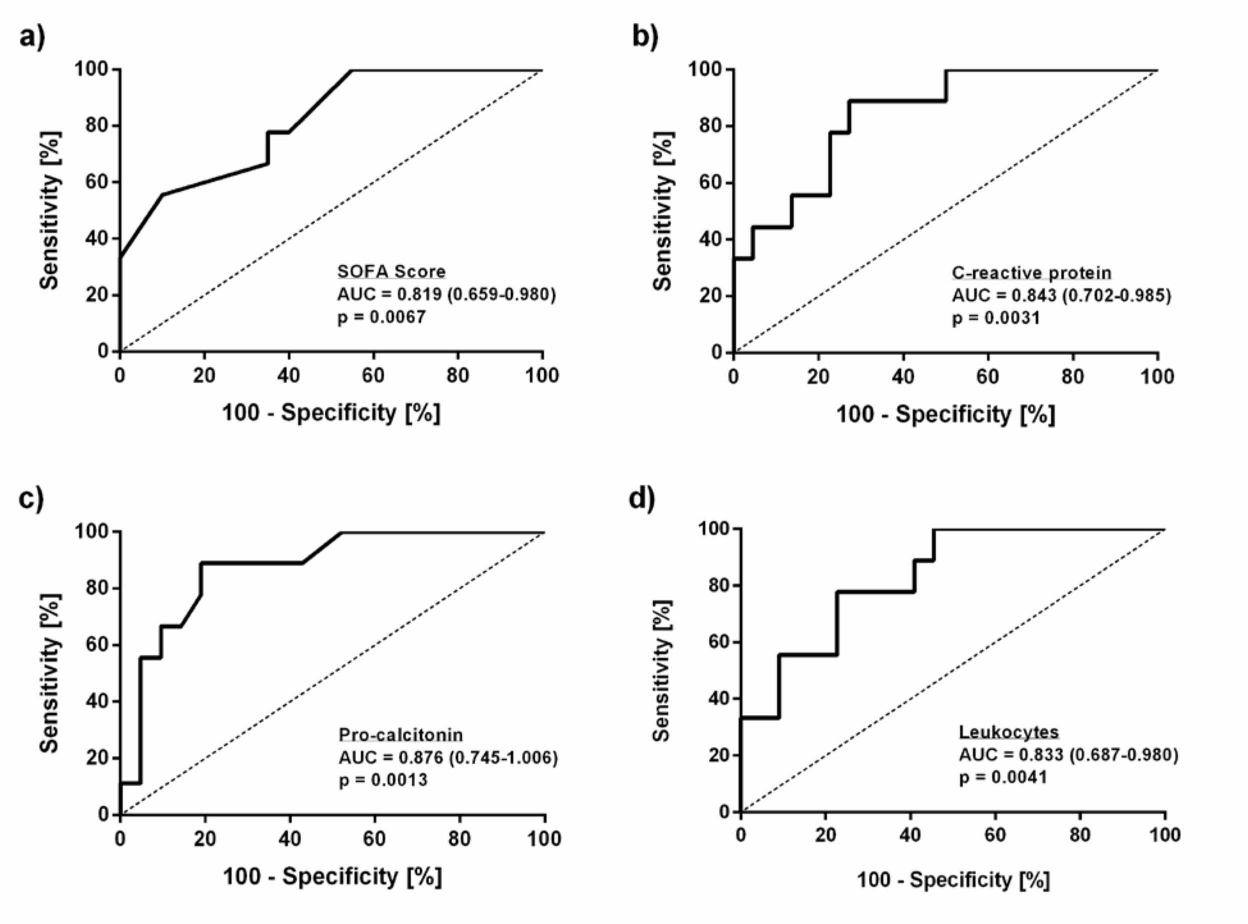

Legend: Receiver-operated curve (ROC) analyses of SOFA score (a), C-reactive protein (CRP) levels (b), pro-calcitonin (PCT) levels (c), and leukocyte cell count (d) for in-hospital mortality of COVID-19 patients. AUC, area under the curve.

Supplementary Figure S7: Kaplan-Meier curves for in-hospital mortality of hospitalized COVID-19 patients stratified for SOFA score and inflammatory markers combined with SDMA and ADMA.

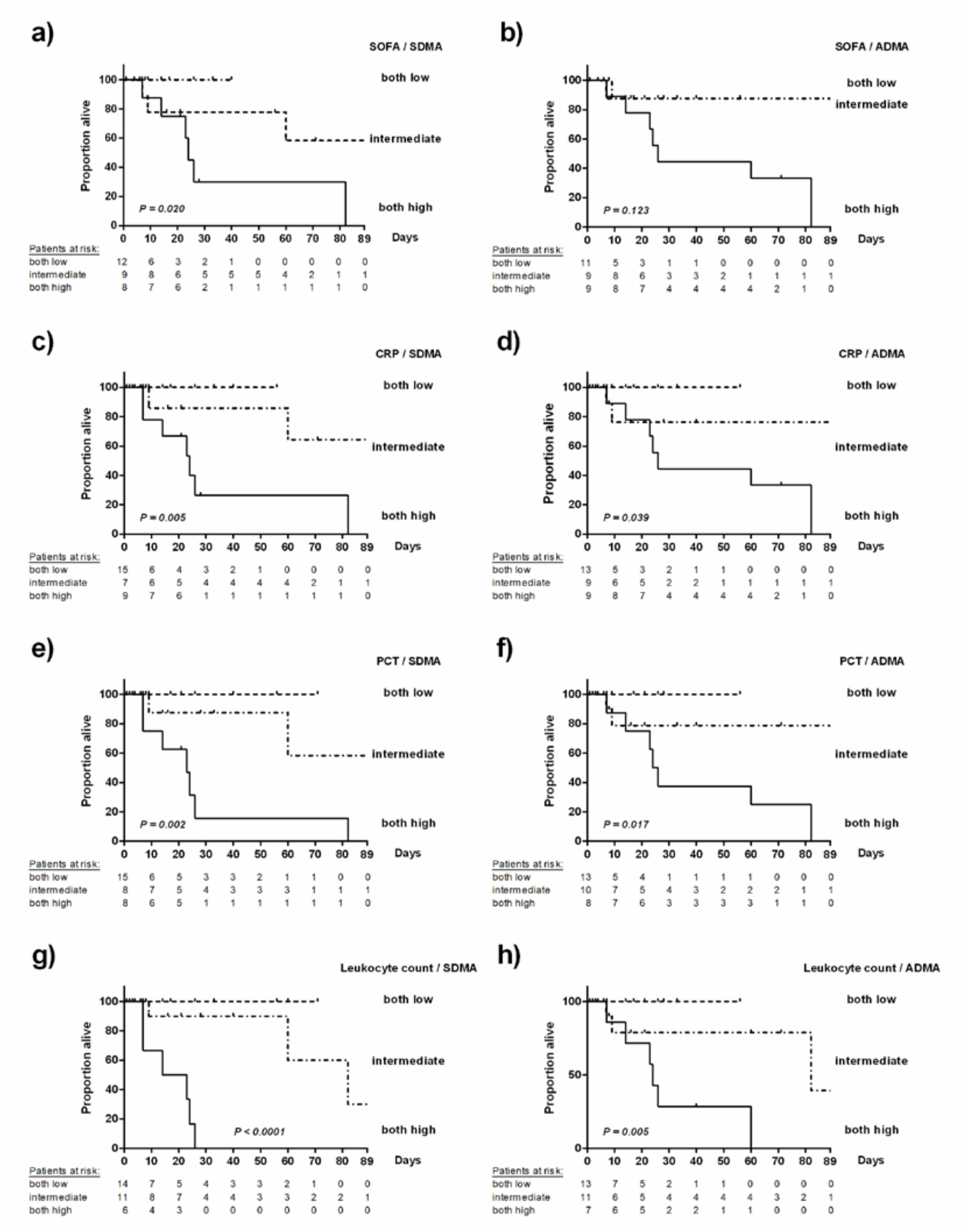

Legend: Kaplan-Meier curves for in-hospital mortality of COVID-19 patients stratified for SOFA score + SDMA (a), SOFA score + ADMA (b), C-reactive protein (CRP) levels + SDMA (c), CRP + ADMA (d), pro-calcitonin (PCT) levels + SDMA (e), PCT + ADMA (f), leukocyte cell count + SDMA (g), and leukocytes + ADMA (h). AUC, area under the curve.

Supplementary Figure S8: Receiver-operated curve (ROC) analyses and Kaplan-Meier curves for in-hospital mortality of hospitalized COVID-19 patients stratified for (SDMA+ADMA) and (SDMAxADMA).

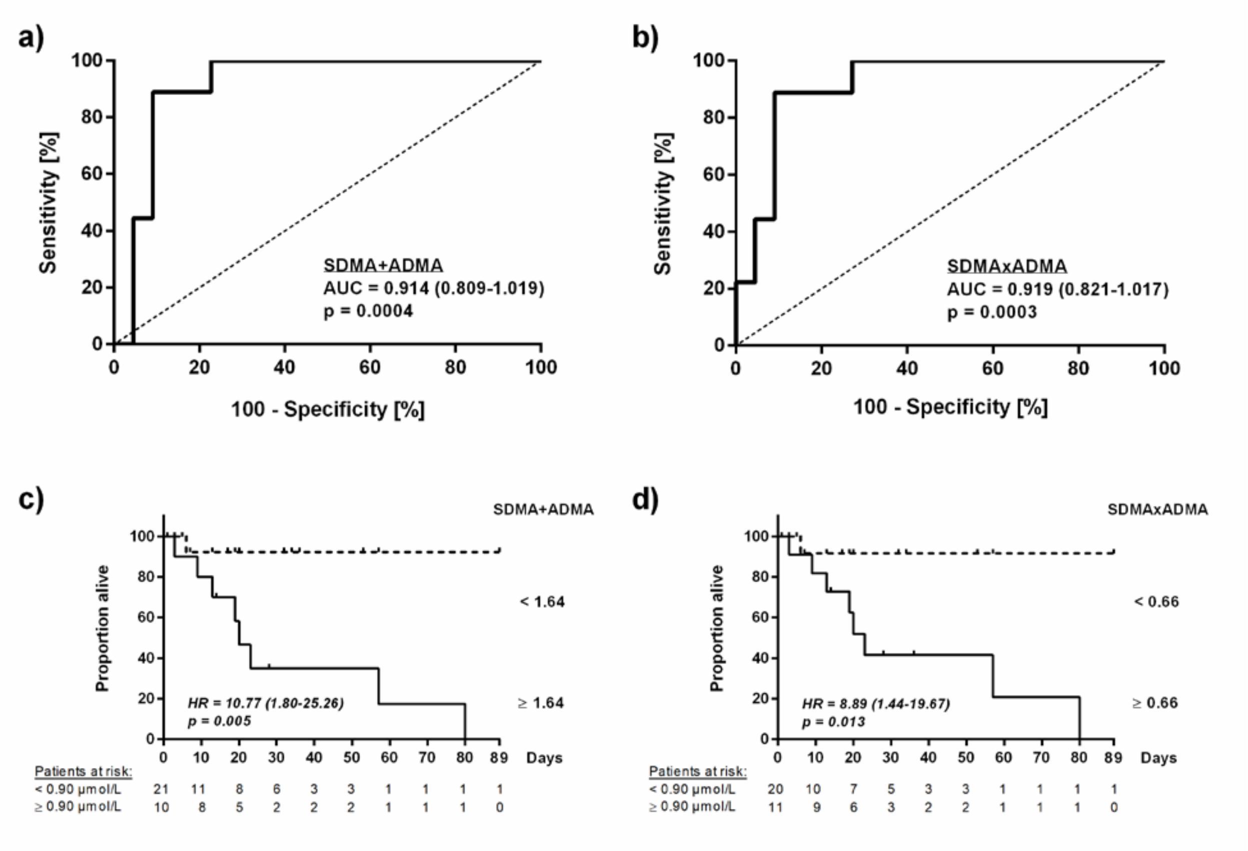

Legend: Receiver-operated curve (ROC) analyses of the combined biomarkers (SDMA+ADMA; a) and (SDMAxADMA; b), and Kaplan-Meier curves for in-hospital mortality of COVID-19 patients stratified for (SDMA+ADMA; c) and (SDMAxADMA; d).

Supplementary Figure S9: Receiver-operated curve (ROC) analyses for previously published COVID-19 mortality risk scores as compared to SDMA and ADMA.

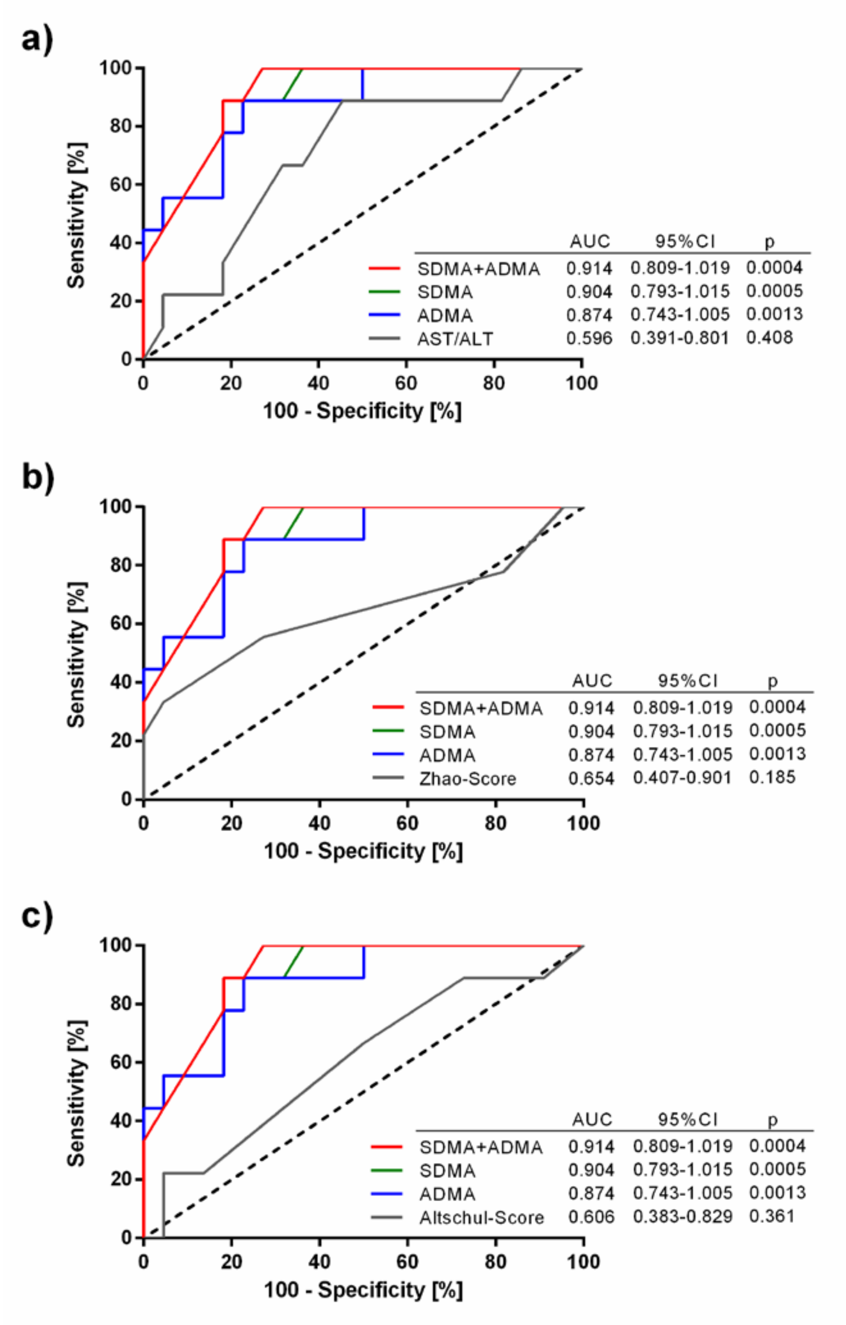

Legend: Receiver-operated curve (ROC) analyses of AST/ALT ratio (a), COVID-19 mortality score published by Zhao and co-workers [8] (b), and COVID-19 mortality score published by Altschul and co-workers [5] (c), each compared to SDMA, ADMA, and (SDMA+ADMA). AUC, area under the curve.
